# Regulation of the two-pore domain potassium channel, THIK-1 and THIK-2, by G protein coupled receptors

**DOI:** 10.1371/journal.pone.0284962

**Published:** 2023-04-26

**Authors:** Michihiro Tateyama, Yoshihiro Kubo

**Affiliations:** 1 Division of Biophysics and Neurobiology, Department of Molecular and Cellular Physiology, National Institute for Physiological Sciences, Okazaki, Japan; 2 Department of Physiological Sciences, School of Life Science, The Graduate University for Advanced Studies (SOKENDAI), Okazaki, Japan; Weizmann Institute of Science, ISRAEL

## Abstract

A member of THIK (two pore domain halothane-inhibited K^+^) channels, THIK-1, was reported as a target of Gi/o-coupled receptors (Gi/o-Rs) in neurons and microglia. We confirmed that in HEK293T cells the THIK-1 channel is activated by Gi/o-Rs and found that Gq-coupled receptors (Gq-Rs) also activates the channel. The effects of Gi/o-Rs and Gq-Rs were inhibited by the Gi/o inhibitor pertussis toxin and phospholipase C (PLC) inhibitor, respectively. The effects of Gi/o-Rs were attenuated when consensus Gβγ binding motif at the C-tail of the THIK-1 channel was mutated, suggesting that Gβγ serves as a THIK-1 channel activator upon the stimulation of Gi/o-Rs. As to the effects of Gq-Rs on the THIK-1 channel, a protein kinase C inhibitor and calcium chelators failed to inhibit the effect of a Gq coupled muscarinic M1R. Neither the hydrolysis of phosphatidyl inositol bisphosphate induced by voltage sensitive phosphatase nor the application of a diacylglycerol analogue, OAG, increased the channel current. The mediator of Gq-dependent activation of the THIK-1 channel remained unsolved. The effects of Gi/o- and Gq-Rs on the THIK-2 channel were also investigated, by using a THIK-2 mutant channel whose N-terminal domain is deleted to improve the surface membrane expression. We observed that Gi/o- and Gq-Rs activate the mutated THIK-2 channel, similarly to the THIK-1 channel. Interestingly, heterodimeric channels of THIK-1 and THIK-2 responded to Gi/o-R and Gq-R stimulation. Taken together, Gi/o- or Gq-Rs activates the THIK-1 and THIK-2 channels in a Gβγ or PLC dependent manner, respectively.

## Introduction

Two-pore-domain potassium (K2P) channels consist of two pore forming domains and four transmembrane (TM) domains. K2P channels dimerize to form a functional channel which constitute background leak K^+^ current [1]. The activity of the K2P channels is reported to be modulated by temperature, pH and mechanical stretch, or regulated by the application of lipids, including arachidonic acid and phosphatidyl bis phosphate (PIP_2_) [1–3]. In addition, G protein coupled receptors (GPCRs) are known as the modulators of several K2P channels, such as TREK-1, TASK, and TASK-3, through changes of their phosphorylation states and/or by the PIP_2_ hydrolysis [4, 5]. The modulation of the K2P channel activity is thought to contribute to the regulation of the excitability of neuronal cells [4, 6, 7].

The THIK-1 (two pore domain halothane-inhibited K^+^) channel is a member of K2P channel family, whose mRNA was shown to express in neuronal cells [8, 9]. The functional THIK-1 channel is expressed in neuronal cells [10–13] and shown in microglia [14–16]. THIK-1 is reported to regulate the microglial raminification and surveillance, and to contribute to the synapse development and the NLRP3 inflammasome activation [14–16]. The THIK-1 channel was also suggested to contribute to the apoptosis through irreversibly increasing its K^+^ conductance by caspase-8 dependent truncation of the C-tail [17]. GPCRs, such as metabotropic GABA receptor (GABA_B_R) or metabotropic glutamate receptor mGlu1, were suggested to increase and decrease the amplitudes of the THIK-1 channel currents, respectively, in cerebellar Purkinje neurons [10]. The effects of Gi/o-coupled receptors (Gi/o-Rs) were shown to be mediated by stimulation of heterotrimeric G protein in Purkinje neurons [12] and to be abolished by pertussis toxin (PTX) in microglia [16]. Recently, PIP_2_ has been reported to directly activate the THIK-1 channel [3], consistently with the inhibitory effect of Gq-coupled mGlu1 [12]. However, the effects of GPCRs on the THIK-1 channel have not been examined in the heterologous expression system. In addition, the effects of activation of GPCRs on the THIK-2 channel, which heterodimerizes with THIK-1 [18], remains unclear.

Here we examined whether or not GPCRs regulate the THIK-1 channel, by recording the macroscopic currents of the THIK-1 channel heterologously expressed in HEK293T cells. We confirmed that Gi/o-Rs activate the THIK-1 channel and found that Gq-coupled receptors (Gq-Rs) positively regulate the channel. Similar responses to activation of Gi/o- and Gq-Rs were observed in the THIK-2 channel as well as the heteromeric THIK-1/THIK-2 channels expressed in CHO-K1 cells, although the current increases differ depending on the compositions of the dimer.

## Materials and methods

### Constructs and expression system

The cDNAs for wild type and mutant THIK-1 channels, mGlu1, KCNQ2 and KCNQ3 were subcloned into pCNX2 vector [12, 19] and cDNAs for receptors, Gβ1 and Gγ2 were into pcDNA3.1(-) expression vector [20–23]. Coding region of mGu2 (M1-P840) was amplified by conventional PCR with designed primers and the resulting product was subcloned into pEYFP-N1 vector at the BglII and EcoRI sites. The cDNAs for mouse THIK-2 channel and dimeric THIK channels were subcloned into pcDNA3.1(-) vector. To increase the surface membrane expression of the THIK-2 channel, 29 residues (Ser2-Ser30) were removed (ΔN-THIK-2) [24]. To construct the homo- or hetero-dimer of THIK channels, THIK-1 and ΔN-THIK-2 channels were ligated in tandem by a long junctional glycine-rich linker (273 amino acid residues), as previously reported [23]. Ci-VSP-IRES-GFP vector was kindly gifted by Dr. Y. Okamura (Osaka University, Osaka, Japan) [25, 26]. HEK293T cells or CHO-K1 cells were seeded on glasses and transfected with the plasmid DNAs for THIK channels, receptors and a transfection marker of yellow fluorescent protein (YFP). Electrophysiological experiments were performed 24–72 h after the transfection. Chelerythrine, GABA, L-glutamate and 1-oleoyle-2-acetyl-sn-glycerol (OAG) were purchased from Sigma-Aldrich (St Louis, MO, USA). 5’-N-ethylcarboxamidoadenosine (NECA), oxotremorine-M (oxo-M), U73122 and U73343 were obtained from Tocris Bioscience (Bristol, UK). Pertussis toxin (PTX) was purchased from Wako (Osaka, Japan). GABA, L-glutamate and oxo-M were dissolved in water, and other reagents were dissolved in DMSO. All reagents were dissolved at high concentration and stored at -20 °C as the stock solutions. The stock reagent solution was diluted at concentrations for the electrophysiological experiments. PTX was solved in the culture medium (final concentration, 300 ng/mL) and cells were incubated in it for more than 16 h. U73122 or U73343 (final concentration, 2 μM each) was dissolved in Hanks’ Balanced Salt Solution (HBSS, Invitrogen, Carlsbad, CA, USA) supplemented with 1 mM Ca^2+^ and 0.3 mM Mg^2+^. Cells were incubated in HBSS with U73122 or U73343 for 10 min at room temperature before the whole cell current recording.

### Electrophysiology

The macroscopic current was recorded from HEK293T or CHO-K1 cells expressing YFP, using whole cell patch clamp technique with Axopatch 200B amplifier, Digidata 1322A and pClamp 9 software (Axon Instruments, Foster City, CA, USA), as previously described [23]. Composition of bath solution was (in mM) 140 NaCl, 4 KCl, 1 CaCl_2_, 0.3 MgCl_2_, 10 HEPES (pH 7.4 adjusted with NaOH). The pipette solution for whole cell patch clamp experiments contained (mM) 130 KCl, 5 Na_2_-ATP, 3 EGTA, 0.1 CaCl_2_, 10 HEPES, 4 MgCl_2_, (pH 7.3 adjusted with KOH). GTP (0.3 mM) was supplemented in the pipette solution. Once the whole cell configuration was established, the cell was held at -80mV and ramp pulses (-120mV to +40mV for 0.4 sec) were applied every 5 sec. Various concentrations of the agonists were applied using a fast perfusion system (VC77SP, Warner Instruments, Hamden, CT, USA) [22]. The PKC inhibitor chelerythrine was included in the pipette solution.

### Statistical analysis

The amplitude of the THIK-1 channel current at 0 mV was measured from every trace induced by the ramp pulse protocol to minimize the fraction of non-selective cationic leak current. The current amplitude before the agonist application was set as the basal current amplitude (I_0_). The effects of GPCRs on the THIK-1 channel were evaluated as the ratio of the current amplitude after the agonist application to I_0_ (I_agonist_/I_0_). The EC_50_ values were estimated by fitting the concentration response curve to a Hill equation (Origin2016; OriginLab, Northampton, MA, USA). In the experiments to analyze the effect of Gs coupled receptor A2aR on the THIK-1 channel, the amplitude at the 10^th^ trace after the application of NECA was normalized to I_0_. The effects of A2aR on the Gi/o-R-induced current increase of the THIK-1 channel was estimated as follows. The amplitude of the current increase at the 15^th^ trace (ΔI_15th_) after the application of GABA or oxo-M were normalized to that at the 5^th^ traces (ΔI_5th_) with or without additional activation of A2aR (ΔI_15th_/ΔI_5th_). All data are shown as means and S.D. The statistical significance between two or more than two groups was estimated by unpaired Student’s *t*-test or by a one-way analysis of variance (ANOVA) followed by Tukey’s test, respectively. To examine whether or not A2aR regulates the THIK-1 channel, the effect of NECA application was statistically estimated by paired Student’s *t*-test. Values of p≤ 0.05 were considered as statistically significant (***:p≤0.001, **:0.001<p≤0.01, *:0.01<p≤0.05, n.s.:p>0.05).

## Results

### Effects of Gi/o- and Gq-Rs on the THIK-1 channel

**Fig 1:**
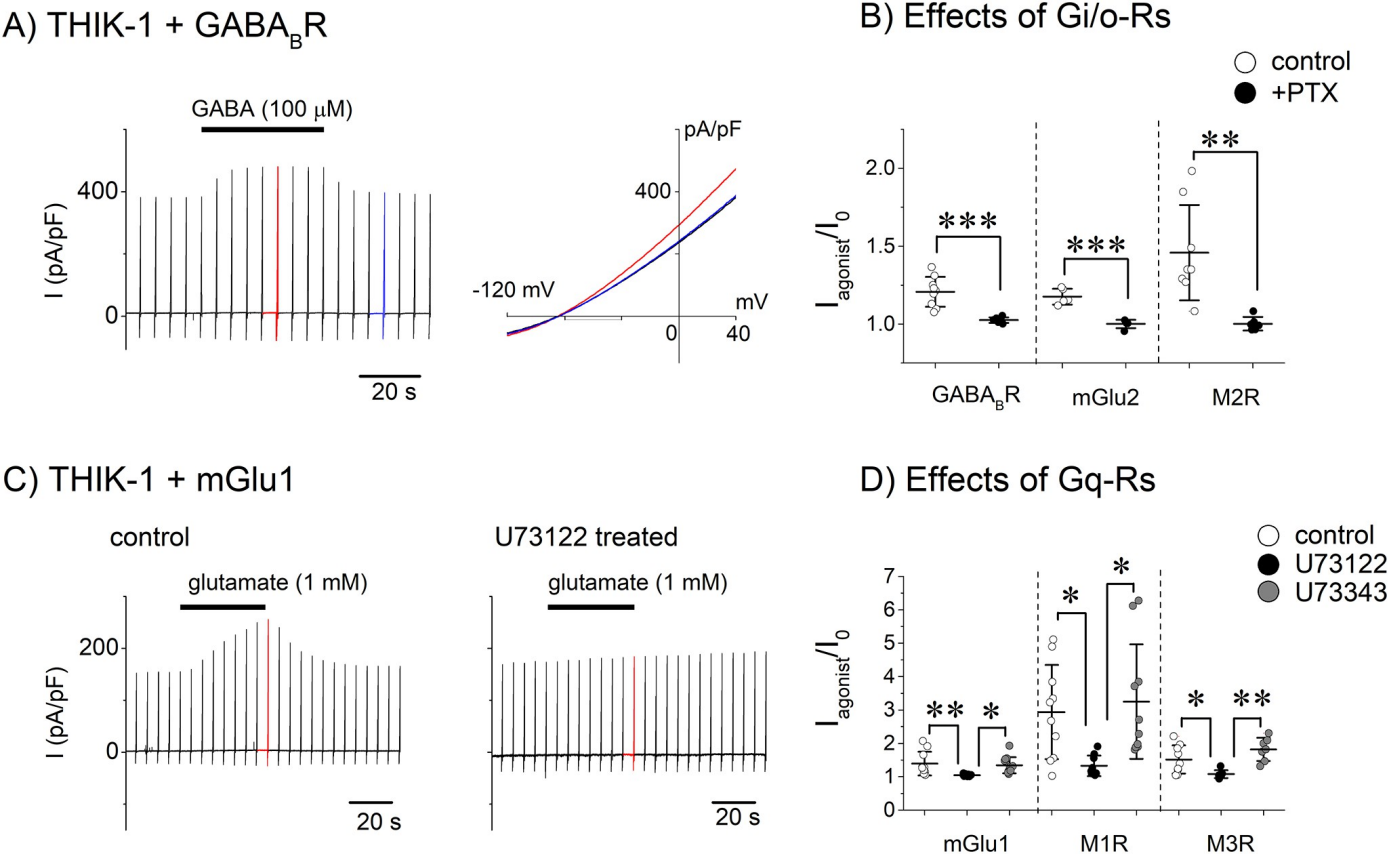
Gi/o- and Gq-coupled receptors increased the amplitudes of the THIK-1 channel current. (A) GABA_B_R activates THIK-1. Ramp pulses from -120 mV to +40 mV (400 ms) were repetitively applied every 5 sec. Shown in left panel is the current trace recorded from a cell co-transfected with THIK-1 and GABA_B_R. The black bar indicates the application timing of 100 μM GABA. The current-voltage relationships before, during and after application of 100 μM GABA (black, red and blue lines, respectively) are shown in the right panel. (B) Effects of Gi/o-Rs. Amplitude at 0 mV of 5^th^ trace after the agonist application was set as I_agonist_ and that before the application was as I_0_. Circles indicate the normalized agonist-induced current amplitude to I_0_ (I_agonist_/I_0_) in each cell without (open circles) or with PTX treatment (filled circles). Mean and S.D. are shown as bars (n = 5–9). Glutamate (200 μM) and oxo-M (10 μM) were applied to activate mGlu2 and M2R, respectively. (C) mGlu1 activates THIK-1 channel. Shown are the current traces recorded from cells co-transfected with THIK-1 and mGlu1 treated without (left) or with (right) U731222 (2 μM, 10 min). Application timing of glutamate (1 mM) is indicated by black bars. (D) Effect of Gq-Rs (I_agonist_/I_0_) in each cell without (open circles) or with treatment of U73122 (filled circles) or U73343 (gray circles). I_agosnist_ was the amplitude of the 7^th^ trace after the agonist application. Oxo-M (10 μM) was applied to activate M1R and M3R. Mean and S.D. are shown as bars (n = 7–12). *: 0.01<p≤ 0.05, **: 0.001<p≤ 0.01, ***: p≤ 0.001. The current densities before application of agonists (I_0_) were not significantly changed by treatments of PTX or PLC inhibitor (see Table 2).

The effect of activation of GABA_B_R on the THIK-1 channel was examined in HEK293T cells. In cells co-transfected with THIK-1 and GABA_B_R, application of GABA (100 μM) increased the current amplitude without changing the reversal potential (Fig 1A). The increase in the current amplitude (I_agonist_/I_0_) was 1.21 ± 0.10 (n = 9, Fig 1B) but not observed when the THIK-1 channel was not co-transfected (S1 Table in S1 File). The effect on the THIK-1 channel was dependent on concentration of GABA (Table 1) and not observed when cells were treated with PTX (300 ng/mL) (Fig 1B filled circles). Similarly, Gi/o-coupled metabotropic glutamate receptor mGlu2 and muscarinic receptor M2R did not change the current amplitude of the endogenously expressed channels in HEK293T cells (S1 Table in S1 File), whereas they increased the amplitude of the THIK-1 channel current, depending on Gi/o and the concentration of the agonists (Fig 1B and Table 1). These results show that Gi/o-Rs activate the THIK-1 channel via stimulating Gi/o in HEK293T cells. Next, we examined the effect of Gq-coupled mGlu1 on the THIK-1 channel, as it was reported that mGlu1 decreased the amplitude of the THIK-1 like current in neuronal cells [10]. Unexpectedly, activation of mGlu1 gradually increased the current amplitude (Fig 1C). The current increase upon the activation of mGlu1 was not observed when the THIK-1 channel was not co-expressed (S1 Table in S1 File), indicating that the increase was derived from the activation of the THIK-1 channel. The effect of mGlu1 was inhibited by the phospholipase C (PLC) inhibitor U73122 (2 μM for 10 min) but not by its inactive form (U73343, 2 μM for 10 min) (Fig 1C and 1D). Activation of Gq-coupled muscarinic M1R and M3R also increased the amplitude of the THIK-1 channel current (Fig 1D) but not of endogenously expressed channels (S1 Table in S1 File). The effects of Gq-Rs were dependent on PLC and the concentration of agonists (Fig 1D and Table 1). PLC is thought to mediate the effects of Gq-Rs on the THIK-1 channel. Taken together, activation of either Gi/o- or Gq-Rs positively regulate the THIK-1 channel via Gi/o or PLC, respectively.

**Table 1:** Efficacy of agonists.

Receptor	agonist	EC_50_	*n*	cells
GABA_B_R	GABA	0.49 ± 0.47 μM	1.23 ± 0.79	3
mGlu2	glutamate	2.83 ± 1.74 μM	1.19 ± 0.65	3
M2R	oxo-M	62.7 ± 35.8 μM	1.54 ± 0.37	4
mGlu1	glutamate	15.0 ± 10.3 μM	1.38 ± 0.47	4
M1R	oxo-M	0.27 ± 0.16 μM	1.14 ± 0.22	3
M3R	oxo-M	0.40 ± 0.31 μM	1.07 ± 0.43	4

Mean and S.D. are shown. *n*; Hill coefficient

The PTX and U73122 treatments did not change the basal current density (I_0_) (Table 2), showing that the spontaneous activity of the receptors in the absence of the agonist is negligible. Interestingly, the averaged I_0_ in cells co-transfected with M1R appeared to be nearly half of those co-transfected with other receptors (Table 2). The small I_0_ in the M1R transfected cells might be a result of M1R-induced unknown mechanisms which decrease surface expression or function of the THIK-1 channel.

**Table 2:** Basal current (I_0_) density of THIK-1 channel.

	control (pA/pF)	+ PTX (pA/pF)	
THIK-1 alone	144.7 ± 88.0 (14)		
THIK-1 + GABA_B_R	145.5 ± 90.2 (9)	170.6 ± 58.2 (7)^n.s.^	
THIK-1 + mGlu2	156.7 ± 72.0 (5)	281.87 ± 148.3 (5)^n.s.^	
THIK-1 + M2R	122.1 ± 48.4 (8)	168.6 ± 141.2 (6)^n.s^	
	control (pA/pF)	+ U73122 (pA/pF)	+ U73343(pA/pF)
THIK-1 + mGlu1	111.2 ± 90.9 (10)	112.2 ± 121.4 (10)^n.s^	121.0 ± 104.7 (12)^n.s^
THIK-1 + M1R	65.2 ± 66.3 (10)	35.7 ± 34.7 (9)^n.s^	46.5 ± 22.3 (10)^n.s^
THIK-1 + M3R	150.2 ± 105.1 (9)	99.2 ± 117.3 (7)^n.s^	113.3 ± 122.1 (7)^n.s^

Basal current density (I_0_) of THIK-1 channel at 0 mV was measured before application of agonists from control cells, PTX treated cells or cells incubated with the PLC reagents. Mean and S.D. are shown. Numbers of experiments are show in parenthesis. n.s.: p>0.05 (vs control)

### Effect of Gs-Rs on the THIK-1 channel

**Fig 2:**
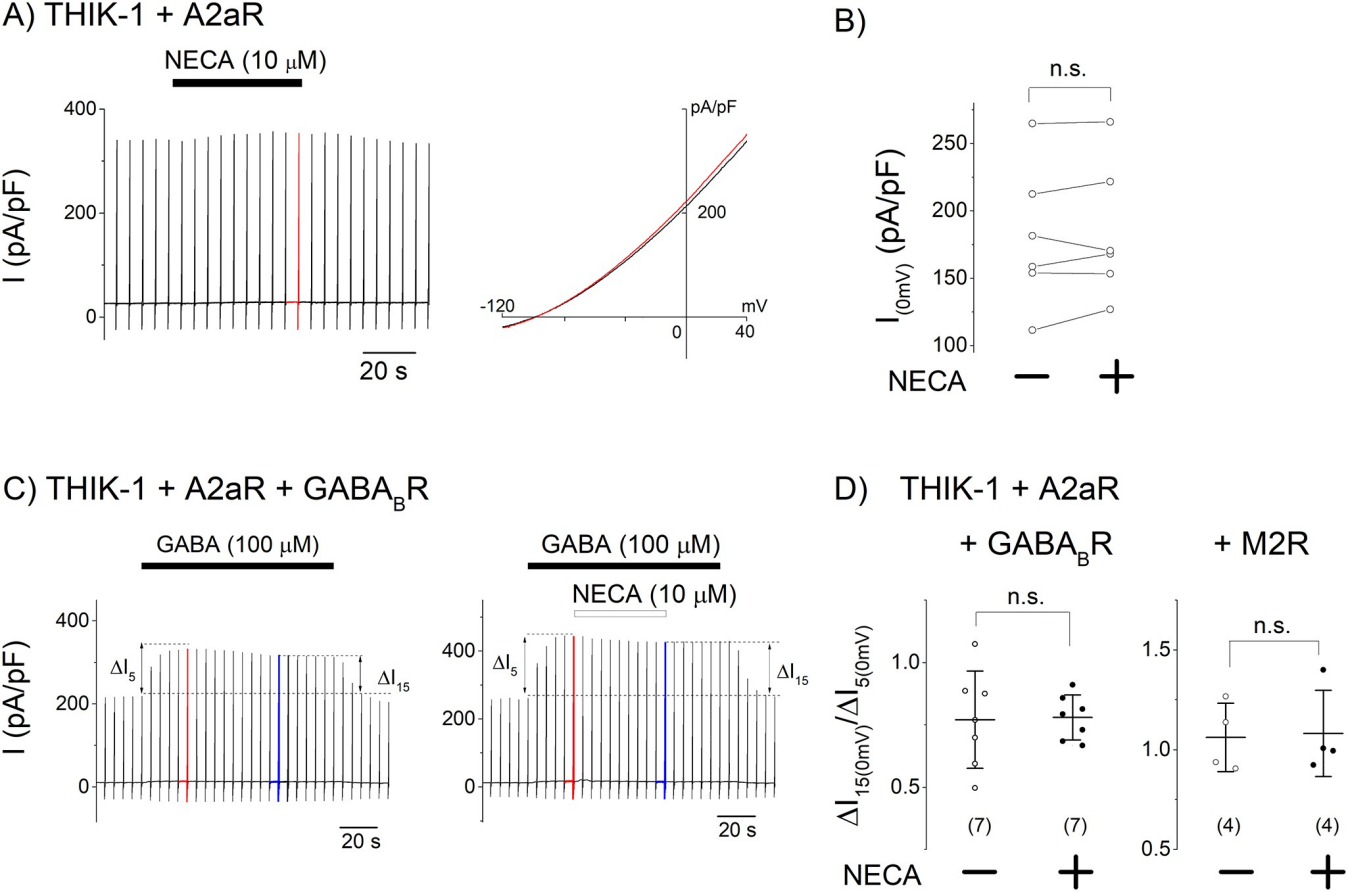
Effects of Gs-coupled A2aR on the THIK-1 channel current. (A) THIK-1 channel current before and after activation of Gs-coupled A2aR. The current trace obtained from the cell co-transfected with THIK-1 and A2aR is shown (left). Applications of 10 μM NECA is indicated by a black bar on the current trace. Shown in the right panel are the current-voltage relationships before and during the NECA application (black and red lines, respectively). (B) Effects of Gs-coupled A2aR on the THIK-1 channel current. Circles indicate the current density before and 50 sec after the application of NECA in each cell (n = 6). (C) Effect of A2aR on the current increase induced by GABA_B_R. The current traces obtained from cells co-transfected with THIK-1, A2aR and GABA_B_R are shown. For control experiment, only GABA (100 μM) was applied (black bars on the traces). For the analysis of the effect of A2aR, NECA (10 μM) was additionally applied (open bar). (D) Effects of A2aR on the Gi/o-R-induced increases in the THIK-1 channel current. Increments of the 15^th^ (blue in C) and 5^th^ current (red in C) amplitudes from I_0_ were measured and ΔI_15th_/ΔI_5th_ was calculated in each cell to evaluate the effect of A2aR. Similar experiments were performed in cells transfected with M2R and A2aR. Oxo-M (10 μM) was applied to activate M2R. Circles indicate the ratios (ΔI_15th_/ΔI_5th_) in each cell without or with additional application of NECA (open or filled circles, respectively). Mean and S.D. are shown as bars (n = 4–7). n.s.: p> 0.05.

Activation of Gi/o inhibits the adenylyl cyclase/PKA signaling and evokes the activation of Gβγ subunits dependent signaling. As PKA dependent phosphorylation is known to inhibit the TREK-1 channel [27], it is possible that inhibition of the PKA signaling potentiates the THIK-1 channel. We examined this possibility by stimulating a Gs-coupled adenosine receptor A2aR, which increased the intracellular concentration of cAMP, a PKA activator [28]. Application of NECA (10 μM) increased the intracellular cAMP in HEK293T cells although the effect was partial (Supporting Materials and Methods, S1 Fig in S1 File), due to the activation of endogenous A2aR (Supporting Materials and Methods, S2 Fig in S1 File). In the cells transfected with heterologous A2aR, Gs signaling was fully activated upon the application of NECA (S1 Fig in S1 File), while the NECA application did not change the amplitude of the THIK-1 channel current (Fig 2A and 2B). The stimulation of A2aR failed to inhibit the effect of GABA_B_R or M2R on the channel (Fig 2C and 2D). These results indicate that Gs signaling did not affect the THIK-1 channel activity, consistently with a previous study in which the PKA activators did not change the THIK-1 channel activity [13].

### Gβγ is a positive regulator of the THIK-1 channel

**Fig 3:**
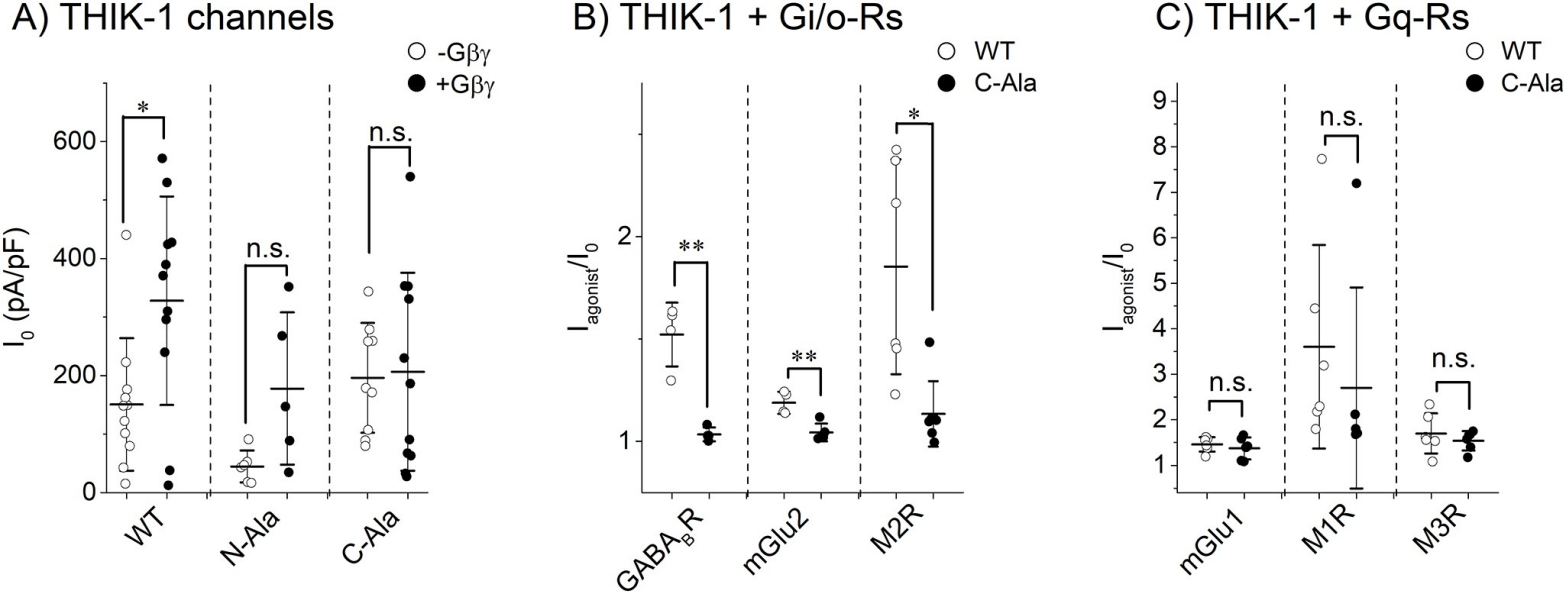
Effects of mutations at the consensus Gβγ binding motif of THIK-1 channel on the current. (A) Effects of Gβγ on the THIK-1 channel. The basal current densities (I_0_) in each cell transfected with wild type THIK-1 channel or the triple mutants (N-Ala; E15A/D16A/N17A, C-Ala; S365A/E366A/M367A) are indicated as open circles. The I_0_ from cells additionally co-transfected with Gβγ, are shown as filled circles. Mean and S.D. are shown as bars (n = 5–11). Co-expression of Gβγ increased the I_0_ of wild type but not of N-Ala and C-Ala mutants. (B) Effects of Gi/o-Rs on wild type and C-Ala THIK-1 channels. Circles show the ratios of the current amplitude after the agonist application to the basal one (I_agonist_/I_0_) in each cell transfected with indicated receptors and wild type THIK-1 channel or C-Ala mutant (open and filled circles, respectively). Mean and S.D. are shown as bars (n = 4–7). (C) Effects of Gq-Rs on wild type and C-Ala THIK-1 channels. Circles show I_agonist_/I_0_ in each cell transfected with indicated receptors and wild type THIK-1 channel or C-Ala mutant (open and filled circles, respectively). Mean and S.D. are shown as bars (n = 6). The receptors were activated by the application of agonists as written in the legend of Fig 1. *: 0.01<p≤ 0.05, **: 0.001<p≤ 0.01, n.s.: p> 0.05.

The effect of Gβγ on the THIK-1 channel was then examined. Co-expression of Gβγ increased the THIK-1 channel current density in the HEK293T cells (Fig 3A), as we previously reported in the study using oocyte expression system [12]. In the previous study, the effect of Gβγ was not observed when a consensus Gβγ binding motif was mutated (N-Ala mutant, E15A/D16A/N17A; C-Ala mutant, S365A/E366A/M367A) [12]. The basal current density of the N-Ala mutant was small when expressed alone (-Gβγ, I_0_ = 44.7 ± 27.3 pA/pF, n = 6), and co-transfection with Gβγ increased the density although the effect was not statistically significant (+Gβγ, I_0_ = 177.9 ± 130.2 pA/pF, n = 5, *p* = 0.083) (Fig 3A). These results suggested that N-terminal domain may have some role in the regulation of the THIK-1 channel activity or surface expression of THIK-1. The basal current density of the C-Ala mutant was not changed by co-expression of Gβγ (-Gβγ, I_0_ = 196.2 ± 93.8 pA/pF, n = 9; +Gβγ, I_0_ = 206.6 ± 169.1 pA/pF, n = 11, with Gβγ, *p* = 0.864) (Fig 3A). The results of C-Ala supported the idea that Gβγ serves as a positive regulator of the THIK-1 channel. In fact, effects of Gi/o-Rs were significantly suppressed in the C-Ala mutant (Fig 3B). In contrast to Gi/o-Rs, the C-Ala mutant responded to the activation of Gq-Rs, similarly to the wild type channel (Fig 3C). From these results, Gβγ was shown as a positive regulator of the THIK-1 channel upon the stimulation of Gi/o-Rs but not of Gq-Rs.

### Effects of a PKC inhibitor, the voltage sensitive phosphatase and the DAG analogue

**Fig 4:**
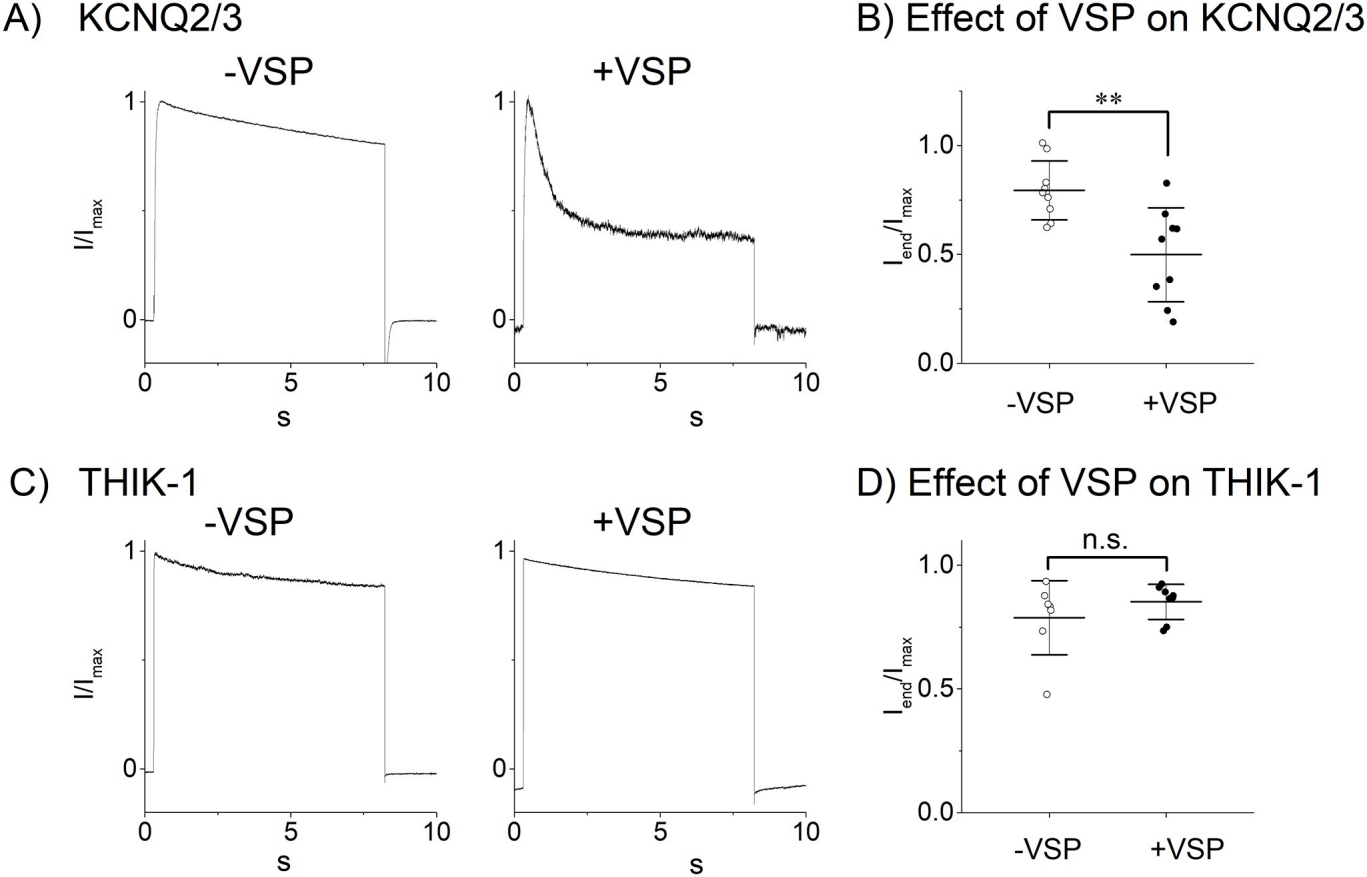
Effect of the PIP_2_ hydrolysis on THIK-1 channel current. (A) Current traces of KCNQ2/3 channels with or without VSP. Cells expressing KCNQ2/3 without (left) or with (right) VSP were depolarized to +40 mV for 8 sec from the holding potential of -80 mV. Traces show the macroscopic currents normalized by the maximal current amplitudes. (B) Effect of the PIP_2_ hydrolysis on KCNQ2/3 channels. Circles show the ratio of the current amplitude at the end of the depolarization to the maximal one (I_end_/I_max_) in each cell without or with VSP (open and filled circles, respectively). Mean and S.D. are shown as bars (n = 9). (C) Current traces of THIK-1 channel with or without VSP. Cells expressing THIK-1 alone (left) or with VSP (right) were depolarized to +40 mV for 8 sec from the holding potential of -80 mV. Traces normalized by the maximal amplitudes are shown. (D) Effect of the PIP_2_ hydrolysis on the THIK-1 channel. Circles show the ratio (I_end_/I_max_) in each cell without or with VSP (open and filled circles, respectively). Mean and S.D. are shown as bars (n = 7–8). **: 0.001<p≤ 0.01, n.s.: p> 0.05.

**Fig 5:**
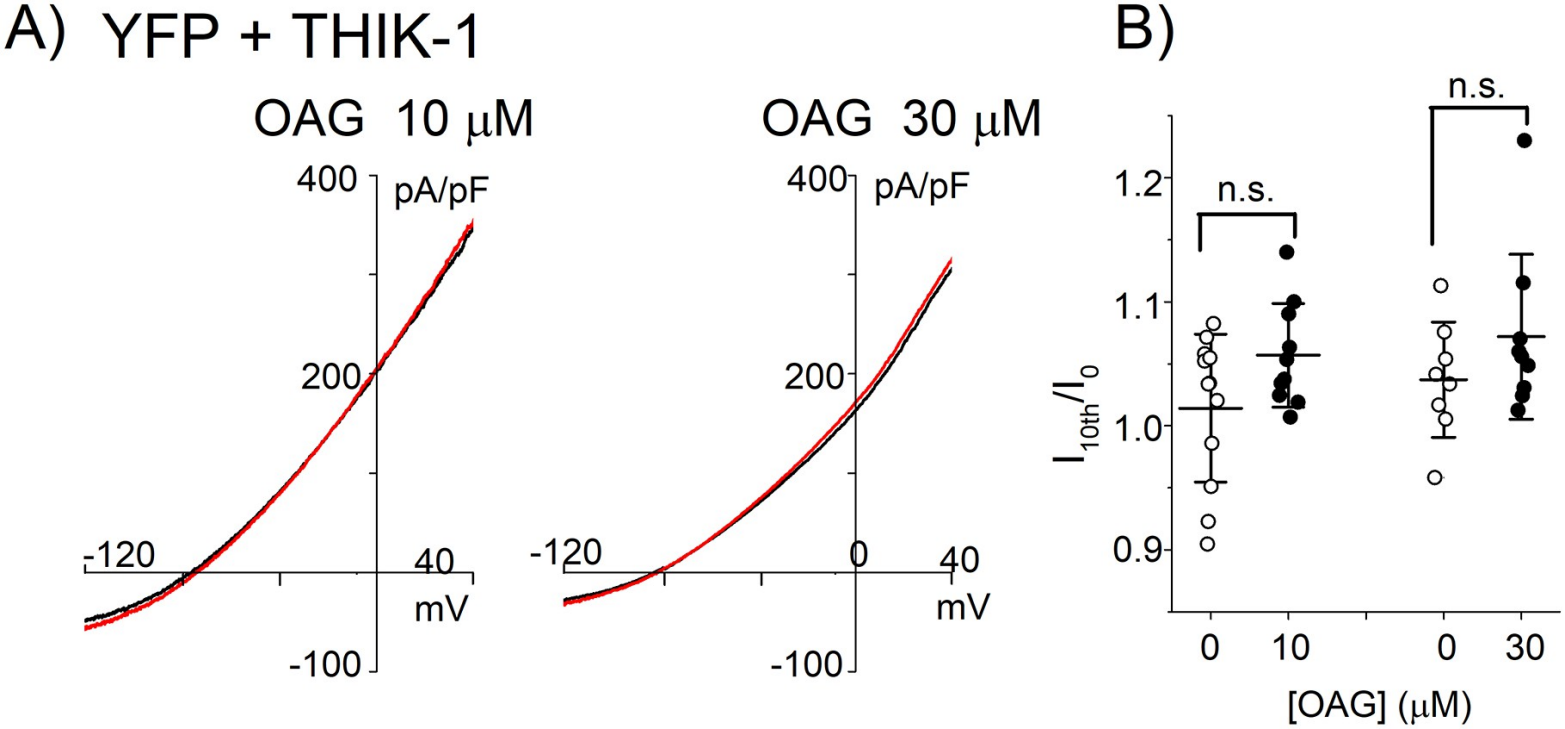
Effects of OAG on THIK-1 channel current. (A) Current and membrane potential relationships before and after application of OAG. Ramp pulses were applied ever 5 sec before and after application of OAG. The current and membrane potential relationships before (black) and 50 sec after application of the indicated concentration of OAG (red) are shown. (B) Effects of OAG on THIK-1 channel. Circles indicate the ratio of the current amplitudes after application of OAG or DMSO to the amplitudes before application of them (I_10th_/I_0_). Mean and S.D. are shown as bars (n = 8–12). n.s.: p> 0.05.

In the present study, a stimulation of Gq-Rs which results in PIP_2_ hydrolysis activated the THIK-1 channel, although PIP_2_ has been recently reported to directly activate the THIK-1 channel [3]. It is possible that Gq signaling molecules, such as inositol tris phosphate, diacylglycerol (DAG) and protein kinase C (PKC) or intracellular free Ca^2+^, exerts potentiating effects on THIK-1 which might be stronger than that of PIP_2_. We then investigated the effects of the Gq downstream signaling molecules. As the pipette solution includes a Ca^2+^ chelator EGTA (3 mM), concentration of the intracellular free Ca^2+^ cannot be increased and therefore Ca^2+^ can be excluded from the candidate. This was confirmed by the result that the effect of the M1R activation was observed even when BAPTA (5 mM) was used (I_agonist_/I_0_ = 3.0 ± 1.0, n = 8). Similarly, inclusion of a PKC inhibitor into the pipette solution (chelerythrin at the concentrations of 3–30 μM) did not inhibit the effect of M1R (Table 3), suggesting that the PKC does not contribute to the increase in THIK channel activity. These results are consistent with the previous study that phorbol myristate acetate did not activate the THIK-1 channel [13]. PIP_2_ is known to interact with and regulate K2P channels [29] and possibly THIK-1 channel [3]. We then examined the effects of a decrease in PIP_2_ on the THIK-1 channel activity, by using the voltage sensitive phosphatase (VSP) which catalyzes PIP_2_ without producing DAG [26, 30, 31]. Application of a depolarization pulse (40 mV, 8 sec) significantly decreased the amplitude of the PIP_2_ sensitive KCNQ2/3 channel current (Fig 4A and 4B), whereas it did not change the THIK-1 channel current (Fig 4C and 4D). The results indicate that PIP_2_ is not involved in the regulation of the THIK-1 channel. Finally, the effect of a DAG analogue, 1-oleoyle-2-acetyl-sn-glycerol (OAG), was examined. An application of the membrane permeable OAG induced a subtle increase in the amplitude of the THIK-1 channel current (Fig 5A red traces), but the increases were not significantly different from those caused by the application of the vehicle (DMSO) (Fig 5B). Taken together, neither PKC, PIP_2_ hydrolysis nor OAG activated the THIK-1 channel. The mediator of the PLC-THIK-1 channel activation remains to be elucidated.

**Table 3:** Effects of PKC inhibitor on M1R-induced potentiation of THIK-1 channel.

THIK-1 & M1R	Basal current I_0_ (pA/pF)	I_oxo_/I_0_	n
chelerythrine 0 μM	38.5 ± 34.7	2.8 ± 1.1	4
chelerythrine 3 μM	43.1 ± 46.8 ^n.s.^	4.4 ± 2.4 ^n.s.^	4
chelerythrine 10 μM	56.4 ± 46.7 ^n.s.^	2.2 ± 0.6 ^n.s.^	3
chelerythrine 30 μM	22.8 ± 15.5 ^n.s.^	2.7 ± 1.0 ^n.s.^	4

The basal current density (I_0_) of THIK-1 channel at 0 mV was measured before the application of oxo-M (10 μM) and the effect of M1R was evaluated as the ratio (I_oxo_/I_0_). The PKC inhibitor, chelerythrine, was dialyzed into cells through the pipette solution. The THIK-1 channel current was recorded five minutes after rupture of membrane. Mean and S.D. values are shown. n.s.: p>0.05 (v.s. 0 μM chelerythrine)

### Effects of mGlu2 and M1R on the THIK-2 channel

**Fig 6:**
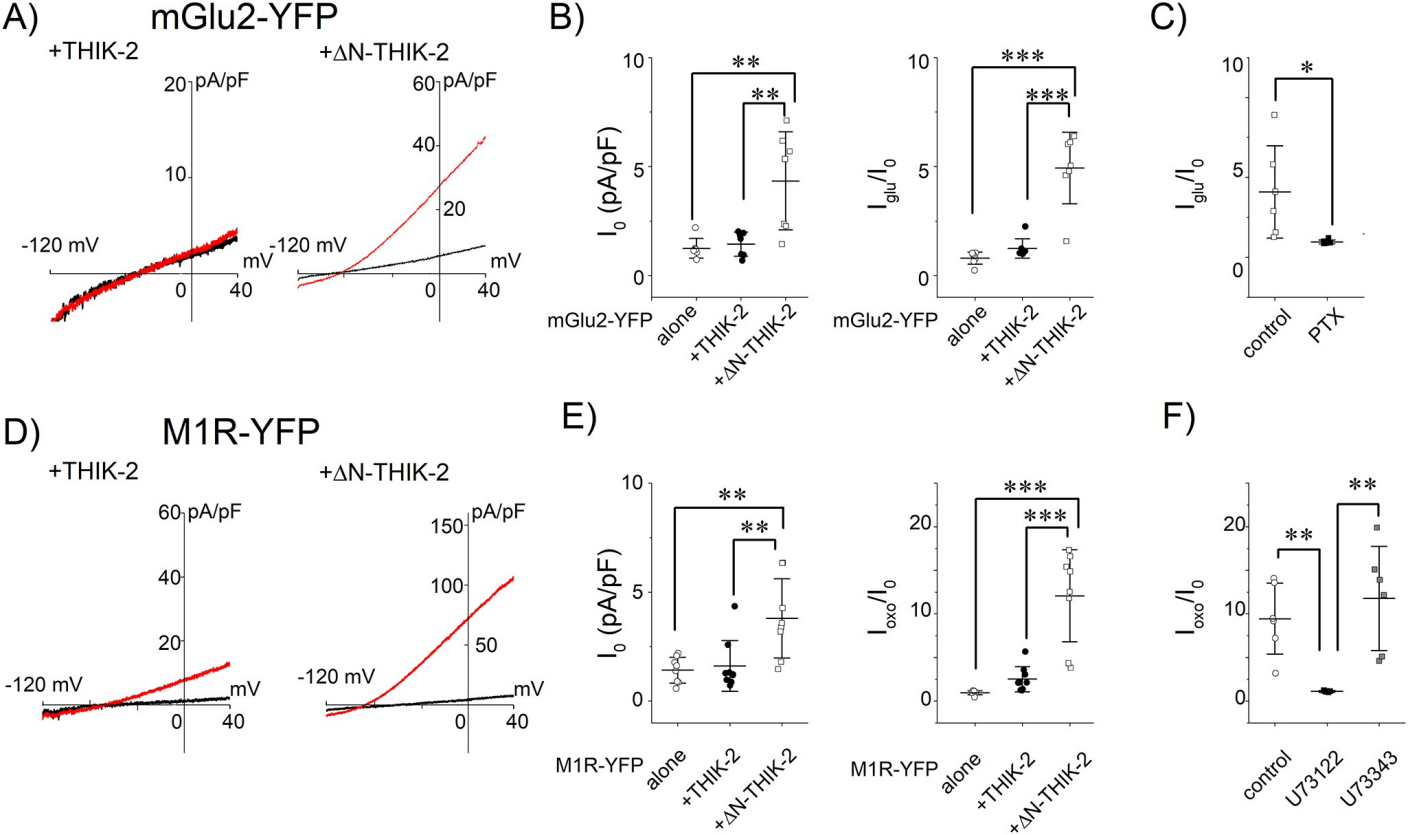
mGlu2 and M1R increased the amplitudes of wild type and ΔN-THIK-2 channel currents. (A) Current-voltage relationships before and after stimulation of mGlu2. Ramp pulses were repetitively applied as written before. Shown are the current-voltage relationships of the THIK-2 channels, before and after application of 200 μM glutamate (black and red lines, respectively). (B) Effects of mGlu2 on the THIK-2 channels. Symbols indicate the basal current densities (I_0_ in left panel) and the effect of the mGlu2 activation (I_glu_/I_0_ in right panel) in cells transfected with mGlu2-YFP alone (open circles), mGlu2-YFP and THIK-2 (filled circle) and mGlu2-YFP and ΔN-THIK-2 (open squares). Mean and S.D. are indicated as bars (n = 7). (C) Effects of PTX treatment on the mGlu2-induced current increase. Open and filled circles indicate I_glu_/I_0_ from control cells and PTX treated cells, respectively. Mean and S.D. are shown as bars (n = 6). (D) Current-voltage relationships before and after stimulation of M1R. Shown are the current-voltage relationships before and after application of 10 μM oxo-M (black and red lines, respectively). (E) Effects of M1R on THIK-2 channels. Symbols indicate I_0_ (left panel) and the effect of the M1R activation (I_oxo_/I_0_ in right panel) in cells transfected with M1R-YFP alone (open circles), M1R-YFP and THIK-2 (filled circle) and M1R-YFP and ΔN-THIK-2 (open squares). Mean and S.D. are shown as bars (n = 8–9). (F) Effect of the PLC inhibitor. The effect of M1R was inhibited by the treatment of cells with U73122 but not with U73343 (filled circles and open squares, respectively). Mean and S.D. are shown as bars (n = 6). *: 0.01<p≤ 0.05, **: 0.001<p≤ 0.01, ***: p≤ 0.001, n.s.: p> 0.05.

THIK-2 is another member of the THIK channel subfamily and their amino acid sequences are highly conserved. We examined the responses of the THIK-2 channel to the stimulation of Gi/o- and Gq-Rs, by using a THIK-2 channel mutant whose amino acid residues at the N-terminal domain is truncated (ΔN-THIK-2) to increase its expression on the plasma membrane [24]. As has been reported [24], the current density of the THIK-2 channel was smaller than that of the ΔN-THIK-2 channel (Fig 6A, 6B, 6D and 6E) when they were expressed in CHO-K1 cells which do not express K^+^ channels endogenously. The effect of Gi/o-Rs or Gq-Rs on the ΔN-THIK-2 channel were examined by co-expressing Gi/o-coupled mGlu2-YFP (S3 Fig in S1 File) or Gq-coupled M1R-YFP [22], respectively. The effect of mGlu2-YFP or M1R-YFP on the ΔN-THIK-2 channel was clearly observed (Fig 6A, 6B, 6D and 6E). As shown in the THIK-1 channel experiments (Fig 1), effects of mGlu2-YFP and M1R-YFP were inhibited by PTX and U73122, respectively (Fig 6C and 6F). Taken together, it was shown that the THIK-2 channel is regulated by Gi/o- and Gq-coupled receptors through the activation of Gi/o and PLC, respectively.

**Fig 7:**
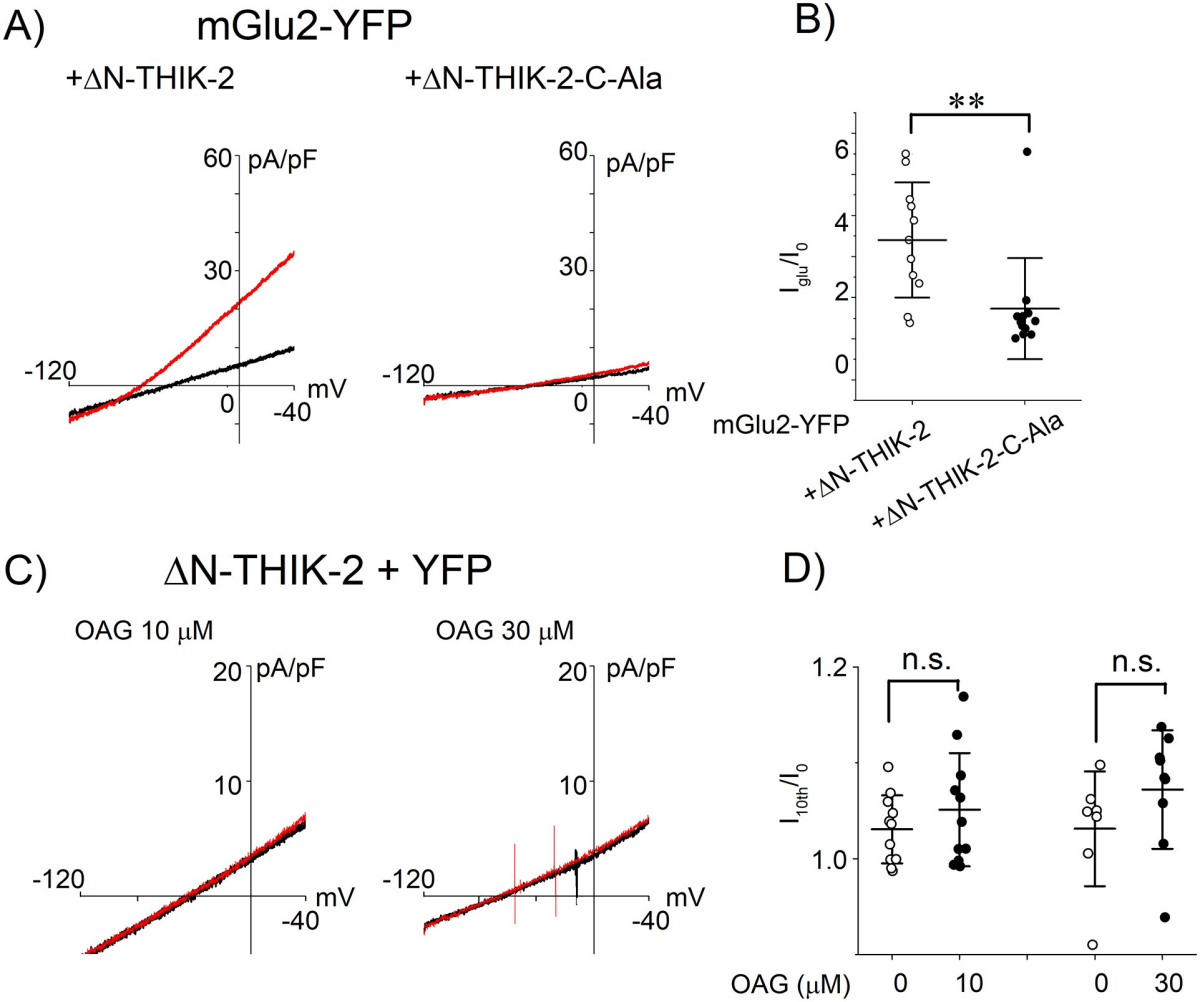
Effect of C-Ala mutation or OAG on ΔN-THIK-2 channel. (A) Current-voltage relationships before and after stimulation of mGlu2. The current-voltage relationships of ΔN-THIK-2 channels are shown before and after application of 200 μM glutamate (black and red lines, respectively). (B) Effects of the mGlu2 activation on the ΔN-THIK-2 and C-Ala mutants. Symbols indicate the potentiating effect of glutamate (I_glu_/I_0_) in each cell transfected with mGlu2 and the ΔN-THIK-2 channel or the C-Ala mutant (open and filled circles, respectively). Mean and S.D. are shown as bars (n = 11–12). (C) Current-voltage relationships before and after application of OAG. The current-voltage relationships before and 50 sec after application of OAG are shown (black and red lines, respectively). The tested concentration of OAG was 10 μM (left) and 30 μM (right). (D) Effects of OAG on the ΔN-THIK-2 channel. The ratio (I_10th_/I_0_) are not different between OAG applied group (filled circles) and vehicle applied group (open circles). Mean and S.D. are shown as bars (n = 7–11). **: 0.001<p≤ 0.01, n.s.: p> 0.05.

As the C-Ala mutation inhibited the response of the THIK-1 channel to Gi/o-Rs (Fig 3), we introduced mutations at the consensus Gβγ binding motif of ΔN-THIK-2 (ΔN-THIK-2-C-Ala, S390A/E391A/T392A). The alanine mutations significantly attenuated the response of ΔN-THIK-2 to mGlu2 (Fig 7A and 7B), suggesting that Gβγ is a regulator of the THIK-2 channel. As shown in Fig 6, the response of ΔN-THIK-2 channel to the M1R activation was much larger than that of the THIK-1 channel (cf. Fig 1D). The ΔN-THIK-2 channel was expected to be highly responsible to the Gq signaling molecules, such as a DAG analogue. However, OAG did not increase the current amplitude at the concentration of 10 and 30 μM (Fig 7C and 7D). The results show that contribution of the DAG analogue to the THIK channel regulation is negligible.

### mGlu2 and M1R activate the heterodimer of the THIK-1 and THIK-2 channel

**Fig 8:**
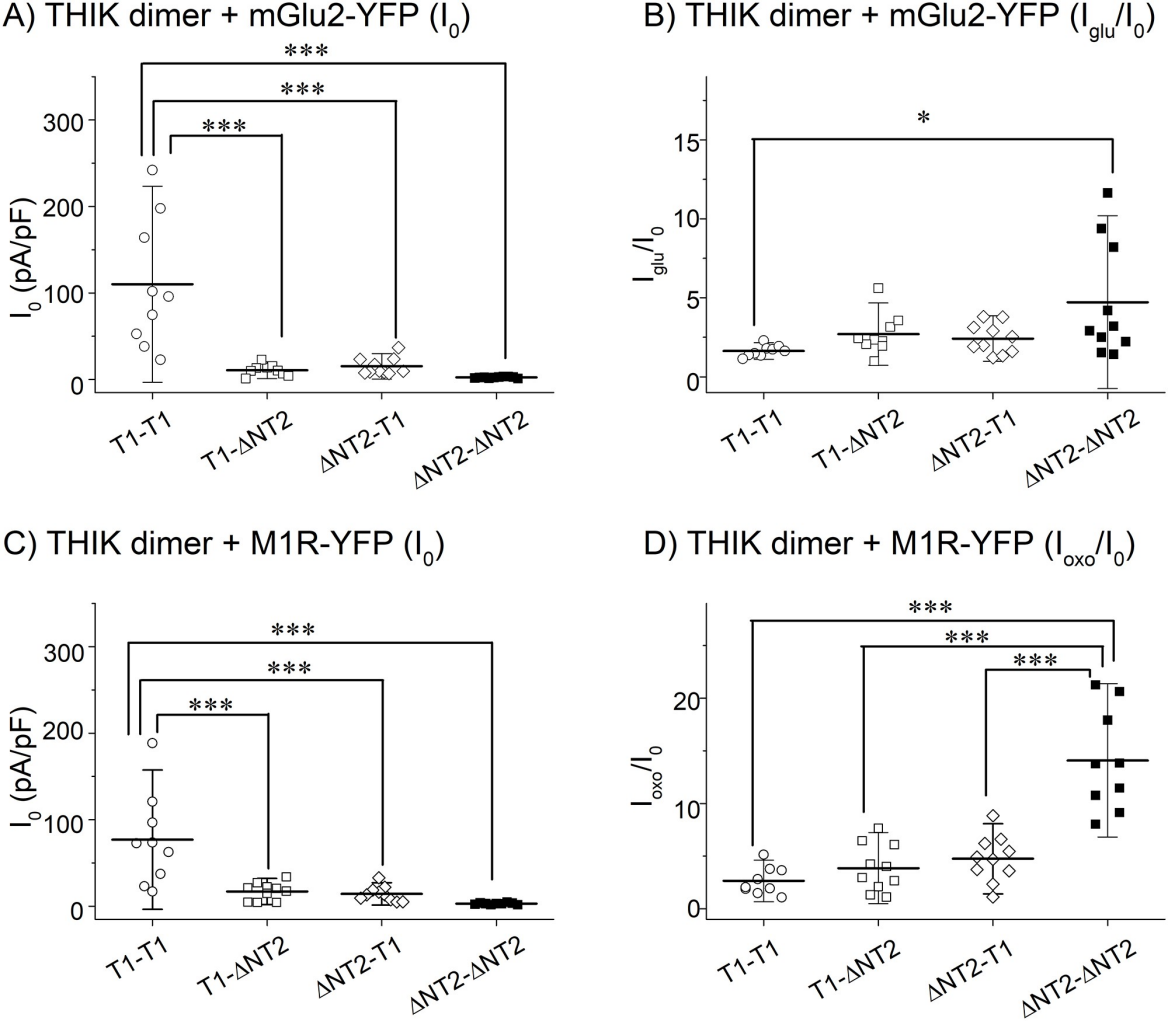
Gi/o- and Gq-R dependent regulation of the heterodimeric THIK channels. (A) Basal current densities of the tandem THIK channels. Symbols show the basal current density (I_0_) of each cell transfected with the indicated dimeric channels. Mean and S.D. are shown as bars (n = 9–10). (B) Effect of mGlu2 on the tandem THIK channels. Symbols show the effects of the mGlu2 activation (I_glu_/I_0_) in each cell transfected with the indicated THIK channel dimers. 200 μM glutamate were applied to activate mGlu2. Mean and S.D. are shown as bars (n = 9–10). (C) Basal current densities of the tandem THIK channels. Symbols show the basal current density (I_0_) of each cell transfected with the indicated dimeric channels. Mean and S.D. are shown as bars (n = 9–10). (D) Effects of M1R on the tandem THIK channels. 10 μM oxo-M were applied to activate M1R. Symbols show effects of the M1R activation (I_oxo_/I_0_) in each cell transfected with the indicated THIK channel dimers. Mean and S.D. are shown as bars (n = 9–10). ***: p≤ 0.001, *: 0.01<p≤ 0.05.

THIK-1 dimerizes with THIK-2 and the heteromeric channel was shown to be functional [18], but it was unclear whether or not the heteromeric channel is activated by GPCRs. To examine the response to the GPCR activation, the THIK channels were ligated by a glycine rich linker (THIK-1/linker/THIK-1, THIK-1/linker/ΔN-THIK-2, ΔN-THIK-2/linker/THIK-1, ΔN-THIK-2/linker/ΔN-THIK-2). As the properties of the homodimers were similar to those of wild type channels, possible influences caused by the ligation and/or the glycine rich linker were negligible. Basal current densities of the heterodimers were between those of the homodimers (Fig 8A and 8C), as shown in the previous study [18]. The extent of the current increases of the heterodimers upon the stimulation of mGlu2 was not significantly different from those of homo-dimers (Fig 8B), whereas those upon the stimulation of M1R were significantly smaller than that of the ΔN-THIK-2 dimer (Fig 8D). These results show that the heterodimer can respond to the Gi/o- and Gq-R activation and suggested that both the basal activity and the enhanced activity by Gq-R were in between those of homodimers.

## Discussion

This study showed that both Gi/o- and Gq-Rs enhance the THIK-1 and THIK-2 channel activity and that Gβγ serves as a regulator upon the activation of Gi/o-Rs. This study also revealed that the heterodimer of THIK-1 and THIK-2 channels is activated either by Gi/o- or Gq-Rs and that its properties, such as the basal current density and the extent of the effect of Gq-R activation, is in between those of THIK-1 and THIK-2 homodimers.

### Positive regulation of THIK-1 and THIK-2 channels by Gi/o- and Gq-Rs

The effects of Gi/o-Rs on THIK-1 channel in HEK293T and CHO-K1 cells are consistent with previous studies using native cells [10, 12, 16], while positive effects of Gq-Rs on the THIK channels are opposite to the results previously reported [3, 10, 13]. In the previous study of neuronal cells [10], it is possible that current derived from non-inactivating K^+^ channels other than THIK-1 was included and inhibited by mGlu1. A negative effect of M1R activation and a positive effect of PIP_2_ were reported on the rat and human THIK-1 channels, respectively [3, 13]. The discrepancy might be caused by a difference in the species of THIK-1 [3, 13], but it is unlikely because the species dependent differences in the sequence are subtle and less than that between THIK-1 and THIK-2 which also responded to the Gq-R stimulation. As polyanionic lipids other than PIP_2_ can regulate the THIK channels [3], possible PIP_2_ depletion might be compensated by unidentified lipids which also confer a basal activity on the THIK channels. The positive regulation of homo- and heteromeric THIK channels by Gq-Rs contrasts with the Gq-R dependent negative regulation of other K2P channels, such as TWIK-1, TREK-1 and TASK [5, 32, 33]. The unique response of THIK may provide the THIK channels with different physiological roles from those of other K2P channels.

### Difference in the response to GPCR between the THIK-1 and THIK-2 channels

The effects of mGlu2 and M1R on the ΔN-THIK-2 channel were more potent than those on the THIK-1 channel (Figs 1 and 6), but I_0_ of ΔN-THIK-2 was much smaller than that of THIK-1 (Table 2 and Fig 6). Because I_0_ of wild type THIK-2 was smaller than that of ΔN-THIK-2 (Fig 6B and 6E), the THIK-2 channel may not have physiological roles in the regulation of the membrane potential, even when THIK-2 channel is activated by Gi/o- or Gq-Rs. In the case of the heterodimeric channel of THIK-1 and THIK-2, THIK-2 is expected to contribute to the hyperpolarization of the membrane potential upon the stimulation of Gi/o- or Gq-Rs, in cells where THIK-1 and THIK-2 are co-expressed, such as the kidney cell [34].

### Possible roles of the C-terminal domain in the regulation of THIK-1 channel

It is known that carboxy-terminal domain (CTD) of the K2P channels plays important roles in the regulation of the channel activity [1]. The proximal region of TREK-1 CTD was suggested to interact with the head groups of the phosphatidyl lipids and thereby to change the channel activity [35]. PKC dependent phosphorylation at the distal CTD of TASK-3 was also shown to suppress the channel activity [36]. Conformational changes in K2P channels, especially at the lower part of the 4^th^ TM connecting to CTD, are thought to couple to the channel activity [35, 37–39]. Recently, a caspase 8-induced cleavage of distal CTD was reported to increase the K^+^ conductance of the THIK-1 channel [17], supporting the negative regulatory role of CTD of the THIK-1 channel. Therefore, it can be speculated that binding of Gβγ to CTD of THIK induces conformational rearrangements which may allow the THIK channel to become more conductive. The mutations at the Gβγ binding motif did not inhibit the effects of Gq-Rs on the THIK channels (Figs 3 and 7), indicating that Gq-R dependent regulatory mechanism is different from the Gi/o-R dependent one. In this study, a PKC inhibitor, PIP_2_ hydrolysis and OAG did not affect the THIK channel activity. It remains unclear which signaling molecules are involved in regulation of the THIK channel activity by Gq-Rs. An identification of the regulators may give a clue to investigate the mechanisms of the Gq-R dependent regulation of the THIK channel.

In conclusion, the THIK-1 and THIK-2 channels are positively regulated either by Gi/o- or Gq-Rs in heterologous expression system and Gβγ is a positive regulator of the THIK channels. The GPCR dependent regulation of the THIK channels may contribute in part to the regulation of the neuronal and glial cells.

## Supporting information

S1 File (DOCX)
