# Supplementary material for: Regulation of the two-pore domain potassium channel, THIK-1 and THIK-2, by G protein coupled receptors
Source: PLoS One. 2023 Apr 26;18(4):e0284962. doi: 10.1371/journal.pone.0284962 (PMC10132538; doi:10.1371/journal.pone.0284962)
Supplement: S1 File — (DOCX) [file pone.0284962.s001.docx]

**Supporting Materials and Methods**

*Analysis of changes in the intracellular cAMP concentration*

HEK293T cells were culture on the glass bottom dishes and transfected with cDNAs for FRET based cAMP indicators [28] and A2aR. 24-48 after transfection, cells were incubated in bath solution containing (mM) 140 NaCl, 1 CaCl_2_, 4 KCl, 0.3 MgCl_2_, 10 HEPES (pH was adjusted to 7.4 with NaOH) and fluorescent signals were acquired as previously described [28]. CFP was excited by 442 nm laser line and emitted light was passed through S470/30 and S535/30 filters (Chroma), respectively. The fluorescence images were then amplified by an image intensifier unit (C8600, Hamamatsu Photonics) and recorded every 5 s using a cooled CCD camera (Qimage Retiga 6000, Roper Scientific). A laser switching controller (Olympus) was operated by using MetaFluor imaging software (Universal Imaging). The fluorescent intensities of CFP and YFP upon the CFP excitation were measured from fluorescent cells and cell free background.

*RT-PCR*

HEK293T cells were cultured in 35 mm dishes at a density of about 70 % and transfected with or without cDNAs for THIK-1, the receptors or Gβ1Gγ2 subunits. 24-48 after transfection, total RNA (tRNA) was isolated from the un-transfected or transfected cells by using NucleoSpin RNA (Takara Bio). The tRNA sample (0.5 μg) in each group was used for RT-PCR by using PrimeScript™ One Step RT-PCR Kit Ver.2 (Takara Bio) and designed primers. The sequences of the designed primers are same as those both of the human and transfected cDNAs. For the PCR reaction, annealing temperature was set at 56 degree and cycle was 28 times, if not noted. Each reactant was analyzed by an electrophoresis in a 0.9 % agarose gel containing ethidium bromide and the image of the gel was acquired by Printgraph (AE6914, Atto) and CCD camera (E-Shot II, Atto). To examine whether or not cDNAs in HEK293T cells are included in tRNA samples, tRNA samples or vector cDNA were used for PCR reaction with PrimeStar Max premix (Takara Bio).

**Table S1 Effect of receptors on the amplitude of the endogenous current**

| Receptor | I_0_ (pA/pF) | Effect of agonist (I_agonist_/I_0_) | cells |
| --- | --- | --- | --- |
| GABA_B_R | 3.8 ± 1.5 | 1.03 ± 0.06 | 4 |
| mGlu2 | 3.8 ± 1.8 | 0.92 ± 0.06 | 4 |
| M2R | 5.4 ± 4.0 | 0.97 ± 0.12 | 4 |
| mGlu1 | 3.7 ± 1.4 | 0.98 ± 0.17 | 4 |
| M1R | 4.7 ± 3.8 | 0.95 ± 0.15 | 3 |
| M3R | 8.3 ± 3.5 | 1.01 ± 0.13 | 4 |

The current density at 0 mV (I_0_) in HEK293T cell transfected with indicated receptors and the effects of the receptor stimulation are shown as Mean and S.D.

**Fig. S1 Effect of NECA application of the Gs signaling in HEK293T cells**

Time dependent changes of the FRET efficiency of the FRET based cAMP indicator (F_FRET_/F_CFP_) are shown. Black bars show the application of the reagents, which was carried out by perfusion of the chamber (0.2-0.3 mL bath volume) with bath solution (1 mL) containing the reagent. The application of 10 μM NECA decreased the FRET value when A2aR was co-transfected (open circles, n= 17 cells), whereas it partial and gradually decreased the value when the A2aR was not co-transfected (closed circles, n= 15 cells). The decrease in FRET were thought to be caused by the activation of the endogenous A2aR (Fig. S2E). Symbols show mean and SD.


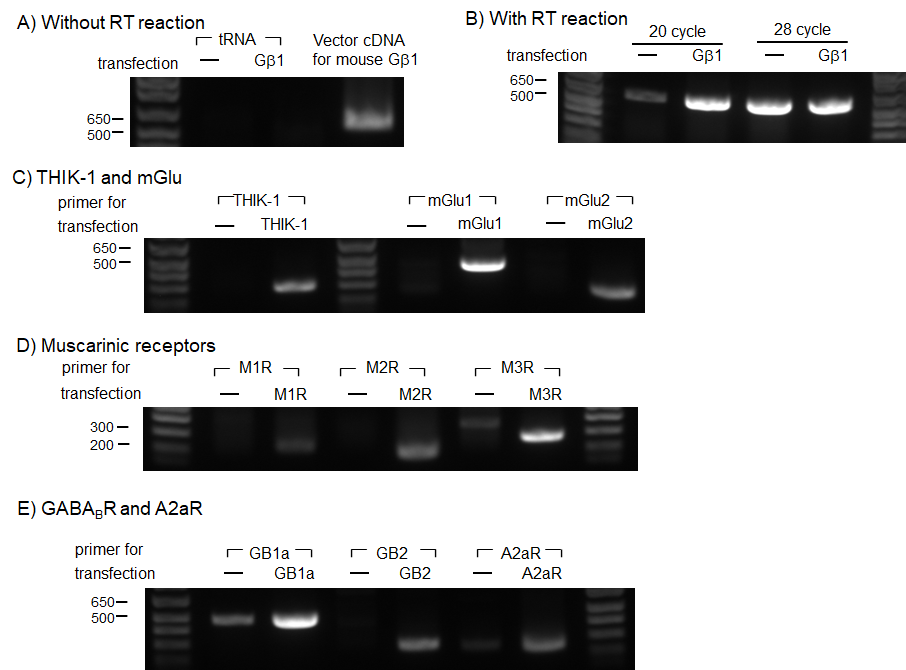


**Fig. S2 Endogenous expression level of THIK-1, receptors and Gβ1**

Results of RT-PCR are shown to estimate the endogenous expression level of the THIK-1 channel, receptors and Gβ1 subunit. A) Results of PCR for Gβ1 in the tRAN samples without RT reaction. The tRNA was collected from cells co-transfected with or without Gβ1. PCR reactants of the indicated tRNA samples or vector cDNA are shown. B) Results of RT-PCR for endogenous and transfected Gβ1 subunit. When the PCR cycle was 20, the band for Gβ1 is faint in the tRNA sample of cells transfected without Gβ1, while it was obvious in tRNA from the cells transfected with Gβ1. The difference is not clear when the PCR cycle was increased. C) Results of RT-PCR for THIK-1 channel, mGlu1 or mGlu2. THIK-1 and mGlus are thought not to express in HEK293T cells. D) Results of RT-PCR for muscarinic receptors. M1, M2 and M3 are thought not to express in HEK293T cells. E) Results of RT-PCR for GABA_B_R and A2aR. GB1a and A2aR, but not GB2, endogenously express in HEK293T cells. As GB1a cannot function as the metabotropic GABA receptor without GB2, possible influence from endogenous GB1a can be ignored. In contrast, the results show that A2aR endogenously expresses in HEK293T cells. This was consistent with the results of the A2aR function shown in Fig. S1.


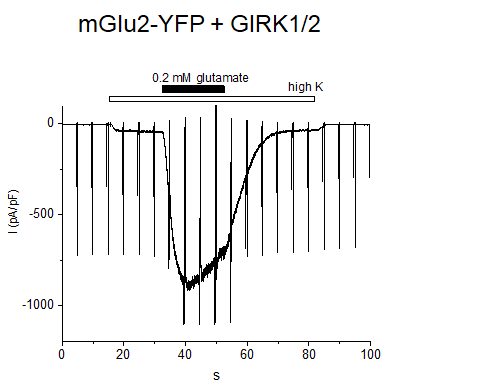


**Fig. S3 mGlu2-YFP activates Gi/o signaling**

The current trace shows the G protein dependent inwardly rectifying K^+^ (GIRK) channel currents recorded from CHO-K1 cells expressing mGlu2-YFP and GIRK1/2 channel [23]. Cells were held at a holding potential of -80 mV and applied ramp pulses at every 5 s, as written in the manuscript. High K^+^ bath solution (white bar on the trace), in which concentrations of KCl and NaCl were exchanged from normal bath solution, was used to facilitate the influx of K^+^ at -80 mV. Application of glutamate (0.2 mM, black bar) evoked the increase in the inward current amplitude at -80 mV; the glutamate-induced increase in the current density was 583 ± 204 pA/pF (3 cells).
